# Treatment of Tonsilitis

**Published:** 1890-04

**Authors:** 


### TREATMENT OF TONSILLITIS.

Dr. Haberkorn (*Centralbl f. Chirurgie*) has employed with excellent results in the different forms of tonsillitis, applications of salicylic acid crystals with the brush to the affected parts. The mucous membrane is covered with the crystals, which dissolve slowly, protecting the healthy tissues and destroying the infectious matter. The applications are made morning and night, and are not attended with discomfort in children over two years of age. Under their use the inflammation rapidly subsides, and the exudations are cast off. If the latter are extensive and thick, they should be previously dissolved by brushing with a solution of pepsine, ʒ ss, muriatic acid, 8 gtt., water, ʒ v, and glycerine, ℥ ss. In addition to the local treatment the author recommends a mixture consisting of acid. salicylic, ʒ ss, solut. gum arabic, ℥ iss, syrup rubi idæi ℥ ss; one tablespoonful every two hours. This mixture reduces the fever, and affords great relief to the local symptoms.

In cases where the inflammation is of a more chronic type, the following mixture administered in tablespoonful doses every three hours is very effective:

| | | |
|---|---|---|
| ℞ | Acid tannic - - - | gr. xv. |
| | Tinct. iodi - - - | gtt. ii. |
| | Aquæ - - - | ℥ vi. |
| | Glycerini - - - | ℥ ss. |

In cases of quinsy the formation of an abscess may be aborted by applying the following:

| | | |
|---|---|---|
| ℞ | Acid tannic - - - | gr. xv. |
| | Tinct. iodi - - - | gtt. ii. |
| | Acid carbolic - - - | ʒ ss. |
| | Aquæ - - - | ℥ iiss. |
| | Glycerine - - - | ℥ ss. |

—*Jour. Respiratory Organs.*

